# Managing bank performance under COVID‐19: A novel inverse DEA efficiency approach

**DOI:** 10.1111/itor.13132

**Published:** 2022-02-24

**Authors:** Sabri Boubaker, Tu D.Q. Le, Thanh Ngo

**Affiliations:** ^1^ EM Normandie Business School Métis Lab France; ^2^ International School Vietnam National University Hanoi Vietnam; ^3^ University of Economics and Law Ho Chi Minh City Vietnam; ^4^ Vietnam National University Ho Chi Minh City Vietnam; ^5^ School of Aviation Massey University Palmerston North New Zealand; ^6^ VNU University of Economics and Business Vietnam National University Hanoi Vietnam

**Keywords:** inverse DEA, Islamic banks, COVID‐19, panel data, efficiency

## Abstract

The evolution of the COVID‐19 pandemic is highly unpredictable; however, its impacts are limited to neither a single sector nor a single country. This study evaluates the performance and efficiency of 49 Islamic banks across 10 countries during 2019–2020 to assess how those banks can preserve their performance and remain resilient in the aftermath of the COVID‐19 pandemic. Using the conventional inverse data envelopment analysis (InvDEA) approach, we show that because of reductions in their outputs, 31 out of the 49 banks studied would need to reduce their inputs so that their efficiency can remain unchanged. However, we show that only 10 banks need to make such adjustments to maintain their efficiency levels using our proposed InvDEA efficiency model. The adjustment for those 10 banks would help in reducing more inputs, suggesting more cost savings, and improving the overall efficiency of the examined banks, compared with the other 31 banks.

## 1 Introduction

The ongoing Coronavirus disease (COVID‐19) pandemic and its worldwide impacts on virtually all areas, such as the healthcare system, international trading, capital and financial markets, and the banking industry, is an event that the world has never witnessed before (Elnahass et al., 2021). International institutions such as the International Monetary Fund (2021) reported a 3.1% drop in the world economic growth rate and an 8.2% drop in global trade volumes for 2020, a situation even worse than the Asian Financial Crisis (AFC) of 1997, the Severe acute respiratory syndrome (SARS) pandemic of 2002–2004, or the Global Financial Crisis (GFC) of 2007–2008. McKibbin and Fernando (2021) found that COVID‐19 caused unprecedented shocks to, among other areas, the labor supply, the equity risk premia of economic sectors, the cost of production, consumption demand, and government expenditure. Similar evidence has been documented for different sectors from different economies (Demirgüç‐Kunt et al., 2021; Elnahass et al., 2021; Rehman et al., 2021).

Banks are the main source of liquidity insurance for numerous economies (Barattieri et al., 2020), and in times of turmoil such as the GFC, it is argued that banks play an especially key role in absorbing shocks (Acharya and Steffen, 2020; Álvarez‐Botas et al. 2021; Demirgüç‐Kunt et al., 2021). Consequently, the resilience of the banking sector is an important driver for the recovery of the global economy (Demirgüç‐Kunt et al., 2021). In this respect, the International Monetary Fund (2021) expects a positive growth rate of 5.9% and 4.9% for the world economy in 2021 and 2022, respectively, given the supportive conditions of the banking and financial sectors. Therefore, the critical requirement for this supporting condition is for the global banking sector to maintain and even improve its efficiency and productivity during and after the COVID‐19 crisis.

The global banking sector consists of two main banking groups: conventional banks (CBs) and Islamic banks (IBs). The former group of banks acts as intermediaries between depositors and borrowers, and thus CBs are expected to transfer the risks between these two players while avoiding supporting risks themselves (Alqahtani et al., 2017; Barattieri et al., 2020). In contrast, the latter group, IBs, operates under the Islamic laws of *Shari'ah*, where the risks are shared among all savers, the IBs, and the borrowers (Olson and Zoubi, 2008; Bourkhis and Nabi, 2013; Barattieri et al., 2020; S&P Global Ratings, 2020). As the Islamic banking industry continues to grow (S&P Global Ratings, 2020), it is undeniable that the IBs are becoming more important to the global banking industry, particularly to the global economic recovery post‐COVID‐19. For instance, Miah et al. (2021) found that Bangladeshi IBs mainly financed trade and commerce investments in 2020, the two sectors that were severely disrupted by the COVID‐19 pandemic. Hassan et al. (2021) observed a similar situation in the Middle East and North Africa (MENA) region. They showed that while IBs in the region have been facing issues due to a fall in oil prices, COVID‐19 has worsened the problems, leaving some banks with literally zero cash inflows. However, other studies such as Beck et al. (2013) and Farooq and Zaheer (2015) have shown that IBs are more resilient to financial shocks (e.g., the AFC or GFC) than CBs. In other words, IBs are expected to recover more quickly than other banks. In line with this reasoning, Elnahass et al. (2021), Rehman et al. (2021), and Demirgüç‐Kunt et al. (2021) have consistently suggested that the IBs were less affected by the pandemic, and thus they responded better to the COVID‐19 crisis than CBs.

It is a common practice to use the frontier analysis approach to evaluate and examine the efficiency and performance of the banking sector by estimating a multi‐dimensional “best practice” frontier for all the banks analyzed, then comparing those banks with that frontier (Hassan and Hussein, 2003; Srairi, 2010; Boubaker et al., 2020). Data envelopment analysis (DEA) and stochastic frontier analysis (SFA) are the two most popular tools used for frontier analysis, with DEA being more popular in the banking efficiency literature because of its flexibility within the complex setting of the banking sector and its relevance to small samples (Liu et al., 2013; Kaffash and Marra, 2017; Boubaker et al., 2018; Emrouznejad and Yang, 2018). More specifically, DEA evaluates the efficiency of the banks by using a given set of inputs to produce a maximum set of outputs (output‐oriented DEA) or by using the minimum set of inputs to produce a given set of outputs (input‐oriented DEA) or a combination of both (Ngo and Le, 2019; Hammami et al., 2020; Boubaker et al., 2021). From a practical perspective, however, one may raise the question of how much input (or output) needs to be consumed (or produced) if a bank wants to reach a certain efficiency level. This is the main question of the traditional inverse DEA (InvDEA), which was proposed and developed by Zhang and Cui (1999) and Wei et al. (2000). Given the impacts of COVID‐19, many banks have witnessed their inputs and especially outputs, such as interest incomes or interest margins, decreasing, so the question of preserving their efficiency as good as it was before COVID‐19 has become important. To date, most InvDEA studies (e.g., Gattoufi et al., 2014; Amin and Ibn Boamah, 2020, 2021) have focused on bank mergers and acquisitions (M&A); none has examined the impacts of such financial shocks, especially on the Islamic banking industry.

The contribution of this study is threefold. First, we examine the impact of COVID‐19 on the performance of 49 IBs across 10 countries by comparing their efficiency between 2019 and 2020. This study has the largest cross‐country sample of IBs so far and is also among the first on the IBs’ performance during the COVID‐19 pandemic. We found that the average efficiency of the sampled IBs increased from 0.613 in 2019 to 0.668 in 2020, supporting the argument that the IBs are more resilient to the financial shock of COVID‐19 (Beck et al., 2013; Elnahass et al., 2021). Second, we show that the impacts of COVID‐19 on individual banks were different, with 31 IBs reducing their outputs but only 10 of them experiencing a drop in their efficiency. This situation justifies our study, as it is the first to use inverse InvDEA to help IBs maintain their efficiency targets, given these real reductions, rather than the use of InvDEA with virtual data in the M&A setting as found in the literature. Third, although traditional InvDEA (based on output reductions) would suggest that these 31 IBs need to adjust their inputs to maintain their performance at the same level as 2019, we proposed an InvDEA based on reduced efficiency (the InvDEAef model), suggesting that only 10 IBs need to do so. The implementation of InvDEAef improved the average efficiency of the IBs in the sample in 2020 to 0.693 (higher than the original value of 0.668) rather than the average efficiency of 0.617 found with InvDEA (even lower than the original value). Therefore, this study is an important improvement for the InvDEA literature and sheds new light on practical ways IBs can efficiently manage their operations under COVID‐19.

The rest of the study is organized as follows. Section 2 reviews the literature on IBs' efficiency and the use of InvDEA in the banking sector. Section 3 explains the relevant methodologies, including DEA, InvDEA, and InvDEAef. Section 4 introduces the data and reports and discusses the empirical findings. Section 5 concludes the study and suggests some directions for future research.

## 2 Literature review

### 2.1 Efficiency of IBs

Previous studies often compare IBs and CBs, with CBs normally treated as the benchmark for IBs. Iqbal (2001) used the (financial) ratio analysis approach to compare the performance of 12 IBs and 12 CBs from 10 countries (including Bahrain, Bangladesh, Egypt, Jordan, Malaysia, Kuwait, Qatar, Saudi Arabia, Turkey, and the United Arab Emirates (UAE) during 1990–1998. The use of ratio analysis was also extended by the work of Hassoune (2002) and Ramlan and Adnan (2016), among others, for evaluating and comparing CBs and IBs. Since ratio analysis could not provide an overall evaluation of bank performance (Song, 2017; Ngo et al., 2019), modern techniques involving multi‐dimensional analyses such as DEA and SFA have been increasingly used in the banking efficiency literature (Shyu et al., 2015; Ngo and Tripe, 2017; Vidal‐García et al., 2018; Daraio et al., 2020). However, these studies have achieved mixed results. Some studies found that CBs outperformed IBs (Hassan, 2006; Kamarudin et al., 2014; Alqahtani et al., 2017; Miah and Uddin, 2017), whereas others suggested that IBs perform better than CBs (Iqbal, 2001; Beck et al., 2013).

According to Miah and Uddin (2017), these mixed findings result from differences in the principles, operations, and characteristics between IBs and CBs. For instance, since IBs operate under Islamic laws (*Shari'ah*), they are only involved in interest‐free financing instruments regarding profit‐and‐loss sharing and markup principles (Olson and Zoubi, 2008; Bourkhis and Nabi, 2013; Ikra et al., 2021; Shah et al., 2021). In this sense, there is evidence that IBs tend to be better capitalized and less risky (Beck et al., 2013; Bourkhis and Nabi, 2013; Majeed and Zainab, 2021), even though their profitability is likely to be lower than that of CBs (Hassan, 2006; Kamarudin et al., 2014; Majeed and Zainab, 2021). Consequently, IBs are less prone to deposit withdrawals when financial shocks happen—they can even attract more deposits during such times thanks to their faith‐based customers (Farooq and Zaheer, 2015). Nevertheless, one may argue that IBs and CBs do not perform with the same efficiency frontier, and thus any comparison between the two needs to be carefully considered. It is therefore justified to carry out evaluations and comparisons among IBs.

Yudistira (2004) was among the first studies applying the DEA to examine the efficiency of 18 IBs in the Middle East and North Africa (MENA) region during 1997–2000. Their study showed that the IBs performed very well during the examined period, with an average efficiency of nearly 90% efficiently. In contrast, IBs outside the Middle East were more efficient than those inside this region. Consistent with Yudistira (2004), Viverita and Skully (2007) also found that Middle Eastern IBs were less productive during 1998–2002 by estimating the total factor productivity of 21 IBs from 13 countries and using Malmquist DEA. In contrast, Tahir and Haron (2010) used SFA to examine the cost and profit efficiency of 193 bank‐year observations of 32 IBs from 2003 to 2008. They found that IBs in the European region were the best performers, whereas those from the Far East and Central Asia were the worst performers. Tahir and Haron (2010) also found that IBs were relatively better at controlling their costs than generating profits, in line with previous findings such as those of Hassan and Hussein (2003) and Kamaruddin et al. (2008). With a longer period covering the 2007 GFC (2007–2012), Bahrini (2017) examined 33 IBs from the Middle East and North Africa using the double‐bootstrap DEA approach. Their main results suggested that the Middle East and North Africa IBs performed well during the GFC. They suggest that these banks should focus more on improving their management practices than increasing their size (Bahrini, 2017).

Interestingly, the argument that IBs are more resilient than CBs to financial shocks (Beck et al., 2013; Farooq and Zaheer, 2015) was examined for the 1997 AFC and the 2007 GFC (Srairi, 2010; Tahir and Haron, 2010; Bourkhis and Nabi, 2013; Rosman et al., 2014); however, there is a lack of studies on the impacts of the recent COVID‐19 pandemic on the performance of IBs. Given that many IBs will have their outputs (e.g., loans, revenues, and profits) reduced because of the pandemic, it is important to analyze their efficiency and provide relevant suggestions for these banks to help them maintain their performance.

### 2.2 InvDEA in the banking efficiency literature

DEA is a non‐parametric approach used to estimate the multi‐dimensional efficiency of homogeneous decision‐making units (DMUs) by using various inputs to produce various outputs (Charnes et al., 1978; Banker et al., 1984). DEA has been extensively used in the banking sector (Emrouznejad and Yang, 2018; Liu et al., 2013; Daraio et al., 2020) because it can flexibly deal with multiple outputs of different natures, and it also does not require an a priori production function; the latter is normally difficult to define clearly for commercial banks (Reinhard et al., 2000; Ngo and Le, 2019). It is worth mentioning that DEA has also been extended to various other fields (e.g., insurance and mutual funds) and different models (e.g., network DEA, Malmquist DEA, bootstrap DEA, and common‐set‐of‐weights DEA) (Simar and Wilson, 2007; Tone and Tsutsui, 2009; Hammami et al., 2020; Ngo and Tsui, 2021).

InvDEA was introduced by Zhang and Cui (1999) to evaluate and manage the investment projects of China's State Economic Information System. Although DEA estimates the (inputs and relative) technical efficiency of each project when the outputs are known, the InvDEA model proposed by Zhang and Cui (1999) estimates the inputs needed for such projects to preserve their DEA efficiency when only the (targeted) outputs are known beforehand. InvDEA was later developed by Wei et al. (2000) in the form of a linear programming problem, for the case of variable returns to scale (Ghiyasi, 2015; Lertworasirikul et al., 2011) and for other different settings (Amin et al., 2017; Amin and Ibn Boamah, 2020; Hadi‐Vencheh et al., 2008).

InvDEA has mostly been applied in the banking sector, with a particular focus on bank M&A (e.g., Gattoufi et al., 2014; Amin and Ibn Boamah, 2020, 2021). In the M&A setting, two or more banks can be merged into a new bank, with the new inputs/outputs being the aggregated value of the independent bank's inputs/outputs before the merge. This situation perfectly matches the idea of InvDEA, where the managers of the new bank need to determine the maximum achievable additional outputs (given the post‐M&A aggregated inputs) or the minimum consumable additional inputs (given the post‐M&A aggregated outputs) to reach a given efficiency target (normally the highest efficiency level of the individual banks before the M&A). For instance, Gattoufi et al. (2014) examined 42 CBs in the Gulf Cooperation Council (GCC) region for 2006 and virtually merged two of them into a new one (for illustration and confidentiality purposes, the authors named them B002 and B003). Their InvDEA results suggested that given the new outputs, to maintain a target efficiency of 0.70 (an arbitrary number), the new bank should keep all the inputs from B002 but reduce the inputs it received from B003. In a similar virtual setting for 28 Canadian banks in 2017, Amin and Ibn Boamah (2020) argued that to improve the efficiency of a post‐M&A bank (generated from banks B04 and B05) from 0.75 to 0.80, the new bank should reduce the deposit inputs from B04 but keep all the deposits inputs from B05; while the labor‐hour inputs should be reduced for both banks. In these M&A studies, we note that the use of virtual data for the post‐M&A banks is an arbitrary detour from the origin of InvDEA. For instance, Wei et al. (2000, p. 162) proposed that the use of InvDEA is to “*estimate the outputs (or inputs) of a DMU from its given inputs (or outputs) by the efficiency index* $z_{0}$ *of the last period*.” Therefore, it is reasonable and advisable to come back to the original idea of InvDEA and examine banks using their observed inputs/outputs and, accordingly, create policy or practical recommendations based on real changes in their inputs/outputs.

It is important to note that most studies have been static (i.e., the InvDEA examination was carried out across different banks but in the same year). Among the few studies in the non‐banking sector, Lim (2016) proposed an InvDEA model to predict future changes in efficiency (based on historical trends) and predict the necessary amount of inputs (outputs) given the efficiency and the amount of outputs (inputs). In this sense, such studies try to answer the question *“Given the expected changes of the efficiency frontier in the future, how can we forecast the inputs (or outputs) needed to maintain the targeted efficiency?*” (Lim, 2016). Despite arguments regarding the accuracy of the predicted score, it is noted that the predicted value is proportional to the observed efficiency. Therefore, the predicted inputs/outputs are also proportions of the observed inputs/outputs, and thus the InvDEA model of Lim (2016) is simply proportional to traditional InvDEA. The idea of Lim (2016) was further extended by Zeinodin and Ghobadi (2019), and this time for bank M&A over different periods. The authors argued that if two banks, B01 and B02, are to be merged, then this should happen in all periods, meaning we should have a merged bank *BM_1_* in Period 1 (merged from B01 and B02 in Period 1) and another merged bank *BM_2_* in Period 2 (merged from B01 and B02 in Period 2), and so on for the other periods. In this sense, one could use InvDEA to compute the optimal inputs (or outputs) of the merged bank *BM* in each period. Although this approach can deal with multiple periods, one can see that for each period, the InvDEA model is not different from traditional InvDEA for bank M&A studies using virtual data.

This study examined the multiple periods. Unlike Lim (2016), we analyzed a panel dataset (of IBs) over 2 years, in which the efficiency scores could be directly measured from the observed inputs/outputs instead of using any predictions or virtual combinations. This led our study back to the basic question of InvDEA, namely, if the inputs (or outputs) of a bank change in Period 2, what will be the optimal outputs (or inputs) to keep that bank as efficient as it was in Period 1? Since we are not dealing with any (virtual) M&A as in Zeinodin and Ghobadi (2019), the results of our analysis are direct and more insightful for managers for setting their targets. More importantly, as explained further in the next section, our InvDEAef model involves data from both periods, reflecting the “dynamic” characteristics of the panel data. By examining the drop in the efficiency of IBs between 2020 and 2019, given the reduction in the bank's outputs due to COVID‐19, this study can suggest the optimal adjustments in terms of their inputs so that their efficiency can be preserved.

## 3 Methodology

### 3.1 Efficiency measurement using DEA

Given the information on multiple inputs and outputs for the set of *n* banks being examined, DEA assigns the optimal weights for the inputs/outputs of a certain bank to those that can bring the bank closest to the frontier, that is, those that can maximize the bank's efficiency. The mathematical expression of DEA, as introduced by Charnes et al. (1978), is:

(1) $$EF_{j_{0}}=\mathrm{ma}x_{u,v}\;\frac{\sum_{r\;=\;1}^{m}u_{r}y_{rj_{0}}}{\sum_{i\;=\;1}^{k}v_{i}x_{ij_{0}}},$$

subject to

$$\frac{\sum_{r}^{m}u_{r}y_{rj}}{\sum_{i}^{k}v_{i}x_{ij}}\;\leq \;1,\;\forall j,\;j\;=\;1,\;2,\;..,\;n$$

$$u_{r},v_{i}\geq \varepsilon ,\;\forall i,r,$$

where $\theta_{j_{0}}$ is the efficiency score of the bank *j_0_* (*j =* 1,2*,..,n*) to be maximized, given the output weight *u_r_* of output *y_r_* (*r =* 1,2*,..,m*), the input weight *v_i_* of input *x_i_* (*i =* 1,2*,..,k*); ε is a non‐Archimedean value designed to enforce positivity on the weights. As discussed in Section 2.2, there have been various improvements and extensions of DEA (e.g., Fujii et al., 2014; Tone and Tsutsui, 2009; Zhu et al., 2019; Ngo and Tsui, 2021), and the readers are therefore encouraged to seek more information from the literature.

For ease of computation, the DEA problem in (1) can be re‐written in a linear programming form as

(2) $$\min\theta_{j_{0},}$$

subject to

$$\sum_{j\;=\;1}^{n}\lambda_{j}y_{rj}\geq y_{rj_{0}},\;r=1,\;2,..,\;m$$

$$\sum_{j\;=\;1}^{n}\lambda_{j}x_{ij}\leq \theta_{j_{0}}x_{ij_{0}},\;i=1,\;2,..,\;k$$

$$\lambda_{j}\geq 0,\;j=1,\;2,..,\;n.$$

### 3.2 InvDEA based on reduced inputs/outputs (the InvDEA model)

In InvDEA, the setting is different from conventional DEA. Given a target efficiency score, the bank is asked to optimize the use of its inputs (or outputs), given a certain change in the outputs (or inputs). This “inverse optimization” problem is the starting point of InvDEA (Zhang and Cui, 1999; Wei et al., 2000). Gattoufi et al. (2014) argued that the input‐oriented InvDEA problem in (3) is an inversion of (2):

(3) $$\min\Delta x_{j_{0}}=\sum_{i=1}^{k}w^{i}\;\Delta x_{ij_{0}},$$

subject to

$$\sum_{j}^{n}\lambda_{j}y_{rj}\geq \left(y_{rj_{0}}+\Delta y_{rj_{0}}\right),\;r=1,\;2,..,\;m$$

$$\sum_{j}^{n}\lambda_{j}x_{ij}\leq \theta_{j_{0}}\left(x_{ij_{0}}+\Delta x_{ij_{0}}\right),\;i=1,\;2,..,\;k$$

$$\lambda_{j}\geq 0,\;j=1,\;2,..,n,$$

where $\theta_{j_{0}}$ is the targeted efficiency score of the bank *j_0_* (*j =* 1,2*,..,n*), $\Delta y_{rj_{0}}$ is the (given) change in its output *y_r_* (*r =* 1,2*,..,m*), $\Delta x_{ij_{0}}$ is the estimated change in its input *x_i_* (*r =* 1,2*,..,m*) and $w^{i}$ is the weight assigned to the inputs $x_{ij_{0}}$ of the bank *j_0_*. In other words, the InvDEA of (3) seeks to minimize the total change in inputs $\Delta x_{j_{0}}$ given certain changes in the outputs $\Delta y_{rj_{0}}$ while preserving a given efficiency score $\theta_{j_{0}}$.

In this sense, the algorithm for (input‐oriented) InvDEA is as follows:

**Step 1**. Compute the DEA efficiency scores for all banks in the sample in each period using Equation (1) or (2).
**Step 2**. Identify the banks for which the outputs changed in the last period, compared with the previous period.
**Step 3**. Use InvDEA as in Equation (3) to estimate the optimal inputs given the observed outputs for each bank identified in Step 2.

### 3.3 InvDEAef model

Equation (3) implicitly assumes that only the banks being examined have their inputs (or outputs) changed (by $\Delta x_{ij_{0}}$ and $\Delta y_{rj_{0}}$) while the other banks and the efficiency frontier stays the same as before the change. The InvDEA estimation of Equation (3) is thus only relevant to that frontier. However, this strict assumption does not hold for multiple periods when the observed frontier may change over time (Lim, 2016; Zeinodin and Ghobadi, 2019). Another issue is that Equation (3) concerns all banks with changes in their outputs (or inputs). In reality, the bank's inputs (or outputs) may also change, such that their efficiency may stay the same or may even increase. In such situations, it would be better to examine only the banks with reduced efficiency between the two periods.^1^ We call this InvDEAef. The InvDEAef algorithm can be expressed as follows:

**Step 1**. Compute the DEA efficiency scores for all banks in the sample in each period using Equation (1) or (2).
**Step 2**. Identify the banks that have reduced their efficiency between two particular periods.
**Step 3**. Use InvDEA as in Equation (3) to estimate the optimal inputs given the observed/targeted outputs for each bank identified in Step 2.
In Step 1 (and Step 3) of this algorithm, it is possible to use any DEA (or InvDEA) model (e.g. Banker et al., 1984; Wei et al., 2000; Ghiyasi, 2015). This study used the basic model of Charnes et al. (1978) because of its simplicity. The main difference between InvDEA and InvDEAef is Step 2. The following empirical section will show that it is an important improvement because InvDEAef can help improve the overall efficiency of the whole sample, although InvDEA cannot.

## 4 Empirical results and discussions

### 4.1 Data on global IBs (2019–2020)

Cross‐country data on the inputs and outputs of the IBs were collected from the Thomson Reuter database. Here, we followed the intermediary approach (Fujii et al., 2014; Hammami et al., 2020) and selected two inputs (operating expenses ($x_{1}$) and total deposits ($x_{2}$)) and two outputs (operating incomes ($y_{1}$) and other earning assets ($y_{2}$)) for our efficiency estimation in Step 1 of InvDEAef. These variables are widely used in the banking efficiency literature, especially in cross‐country settings (Hassan, 2006; Rosman et al., 2014; Ngo and Tripe, 2017; Amin and Ibn Boamah, 2020). The data for 2019 and 2020 were chosen to examine the impacts of COVID‐19 on the sampled banks. Accordingly, our data cover 49 IBs across 10 countries (Bangladesh, Bahrain, Egypt, Jordan, Kuwait, Oman, Pakistan, Qatar, Sri Lanka, and the UAE). Table 1 presents the descriptive statistics of our inputs and outputs data (in billion US$, 2010 constant prices), in which one can see a mix of changes happening to the bank's inputs and outputs. For instance, the average operating expenses ($x_{1}$) dropped from $9.38 billion in 2019 to $8.26 billion in 2020, but the deposits ($x_{2}$) increased from $265.48 billion to $301.83 billion for the same period. On the other hand, fluctuations in those inputs across the sampled banks became larger, with the values of the standard deviation for $x_{1}$ being $12.46 billion and $11.05 billion, but the same figures for $x_{2}$ being $338.48 billion and $397.66 billion, respectively, for 2019 and 2020. A similar situation was observed for the outputs, justifying the use of InvDEA.

**Table 1:** Descriptive statistics for the inputs and outputs of the sampled Islamic banks (IBs) (2019–2020)

		Mean	Standard deviation	Minimum	Maximum
***2019***	$x_{1}$	9.38	12.46	0.01	46.46
	$x_{2}$	265.48	338.48	0.46	1558.00
	$y_{1}$	32.56	42.78	0.06	181.61
	$y_{2}$	126.84	204.56	0.26	970.31
***2020***	$x_{1}$	8.26	11.05	0.00	44.20
	$x_{2}$	301.83	397.66	0.57	1764.39
	$y_{1}$	32.57	43.23	0.06	175.43
	$y_{2}$	168.92	271.14	0.35	1242.77
***Change***	$\Delta x_{1}$	–1.12	–1.40	0.00	–2.26
	$\Delta x_{2}$	36.35	59.18	0.11	206.40
	$\Delta y_{1}$	0.01	0.45	0.00	–6.18
	$\Delta y_{2}$	42.07	66.57	0.09	272.46

*Note*: This table provides information on the mean, standard deviation, minimum and maximum values of our input and output variables for the 49 IBs involved in our research for 2019 and 2020 and the changes between the 2 years. The two inputs are *Operating Expenses* ($x_{1}$) and *Total Deposits* ($x_{2}$) while the two outputs are *Operating Incomes* ($y_{1}$) and *Other Earning Assets* ($y_{2}$). All units are in billion US$ (2010 constant prices).

### 4.2 DEA efficiency of the global IBs

**Fig. 1:**
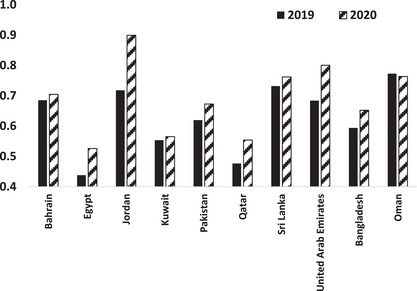
Data Envelopment Analysis (DEA) efficiency scores of the examined Islamic banks (IBs) (by country). This figure shows the country average efficiency scores of the IBs for 2019 and 2020, with higher scores indicating better performance.

We computed the efficiency of the sampled 49 IBs for 2019 and 2020 as in Step 1 of our InvDEAef algorithm using the Solver optimizer function of MS Excel (Fylstra et al., 1998). Interestingly, we found that the average efficiency of these IBs increased from 0.627 in 2019 to 0.690 in 2020, with most countries experiencing some efficiency improvements for their IBs (see Fig. 1). Among the countries involved, the IBs from Jordan and the UAE had the largest improvement, with Jordanian banks having their average efficiency scores increased from 0.718 in 2019 to 0.899 in 2020 while that of UAE's banks increased from 0.684 to 0.801. In contrast, the IBs from Oman faced the largest reduction in performance, and dropped from an average of 0.773 in 2019 to 0.763 in 2020—the latter issue was documented in KPMG (2020) and Mihajat (2021), among others.

**Fig. 2:**
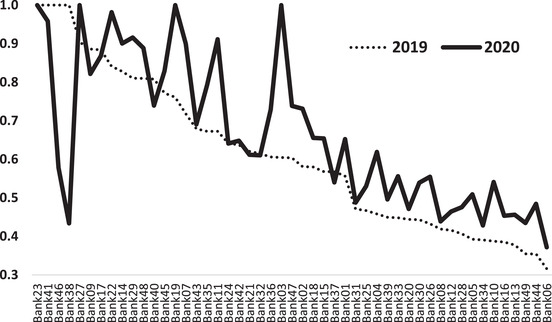
DEA efficiency frontier in 2019 and 2020. This figure shows the efficiency scores of the individual banks for 2019 (the dotted line) and 2020 (the solid line), with higher scores indicating better performance.

At individual banks, Fig. 2 shows that the 2020 efficiency frontier (i.e., the solid line) rises above the 2019 efficiency frontier (i.e., the dotted line) for most of the examined IBs (see also Table 2). This suggests that the performance of the Islamic banking industry improved even under the impacts of COVID‐19. This finding, however, is not surprising but strengthens the argument that IBs are more resilient than CBs when dealing with periods of turmoil (Beck et al., 2013; Bourkhis and Nabi, 2013; Farooq and Zaheer, 2015; Miah and Uddin, 2017). It is also consistent with the recent findings of Elnahass et al. (2021), in which the negative effects of COVID‐19 were found to be much lower for IBs than for CBs. Rehman et al. (2021) also suggested that IBs responded better to the COVID‐19 crisis than CBs.

**Table 2:** IBs need to adjust their inputs according to inverse DEA (InvDEA) and InvDEA based on reduced efficiency (InvDEAef)

Decision‐making unit (DMU)	Drop in outputs	Drop in efficiency	DMU	Drop in outputs	Drop in efficiency
***Bank01***	Yes	No	***Bank26***	Yes	No
***Bank02***	Yes	No	***Bank27***	Yes	No
***Bank03***	Yes	No	***Bank28***	Yes	No
***Bank04***	Yes	No	***Bank29***	Yes	No
***Bank05***	Yes	No	***Bank30***	Yes	No
***Bank06***	Yes	No	***Bank31***	Yes	No
***Bank07***	Yes	No	***Bank32***	Yes	Yes
***Bank08***	Yes	No	***Bank33***	Yes	No
***Bank09***	Yes	Yes	***Bank34***	Yes	No
***Bank10***	No	No	***Bank35***	No	No
***Bank11***	No	No	***Bank36***	Yes	No
***Bank12***	No	No	***Bank37***	Yes	Yes
***Bank13***	No	No	***Bank38***	No	Yes
***Bank14***	No	No	***Bank39***	No	No
***Bank15***	No	No	***Bank40***	Yes	Yes
***Bank16***	Yes	No	***Bank41***	Yes	Yes
***Bank17***	Yes	Yes	***Bank42***	Yes	No
***Bank18***	No	No	***Bank43***	Yes	No
***Bank19***	Yes	No	***Bank44***	Yes	No
***Bank20***	Yes	No	***Bank45***	No	No
***Bank21***	No	Yes	***Bank46***	No	Yes
***Bank22***	Yes	No	***Bank47***	Yes	No
***Bank23***	No	No	***Bank48***	No	No
***Bank24***	No	Yes	***Bank49***	No	No
***Bank25***	No	No			
**Identified by InvDEA (output drop): 31**					
**Identified by InvDEAef (efficiency drop): 10**					

*Note*: This table shows a comparison between 2020 and 2019 for the 49 IBs involved in our study regarding whether a certain bank had a drop in any of its outputs or a decrease in its efficiency score. The former cases are identified as banks that need to adjust their inputs according to the InvDEA approach, whereas the latter are the targets of the InvDEAef approach.

Despite those overall improvements, some variation appeared for individual banks over the 2 years (2019 and 2020). As expected from the pandemic and as observed in Table 2, the reductions in outputs required some IBs to revise their inputs accordingly to preserve their performance. Following Step 2 of the traditional InvDEA approach, one can identify 31 banks with at least one output reduced in 2020, compared with 2019. Accordingly, they all need to adjust their inputs to maintain their efficiency as high as they were in 2019. However, Step 2 of the InvDEAef algorithm suggests that adjustments are necessary for only 10 IBs (see Table 2).

### 4.3 Comparison between the results of InvDEA and InvDEAef

We present the estimated results of InvDEA and InvDEAef, in terms of the efficiency of the IBs and their adjusted inputs, in Table 3. Two important findings can be drawn from the comparison in Table 3. First, for the targeted year of 2020, although InvDEAef helped improve the overall (average) efficiency of the whole sample from 0.690 (Column 4) to 0.715 (Column 6), the InvDEA results showing an average efficiency of 0.631 (Column 5) showed an improvement over the 2019 value (Column 3) but a decrease, compared with 2020. Therefore, we argue that the InvDEAef approach helps preserve the efficiency scores, which is the core value of InvDEA and improves this measurement. Second, according to the InvDEA approach, 31 IBs involved could save a total of $562 billion in operating expenses (*x_1_*) and $8666 billion in deposits (*x_2_*). In contrast, according to the InvDEAef approach, 10 IBs could save $432 and $13,136 billion in terms of the two inputs. Consequently, we argue that the InvDEAef approach is practically more efficient than the traditional InvDEA in terms of cost savings, given that lowering the inputs would help minimize the costs of the banks. These savings are more important, considering the adverse impacts of COVID‐19. As such, bank managers would be better off using InvDEAef for making their decisions than using InvDEA.

**Table 3:** Comparison between InvDEA and InvDEAef for IBs (by country)

				Results of InvDEA				Results of InvDEAef			
Country	Number of banks	DEA efficiency 2019	DEA efficiency 2020	Revised banks	New efficiency	$\Delta x_{1}$	$\Delta x_{2}$	Revised banks	New efficiency	$\Delta x_{1}$	$\Delta x_{2}$
***Bahrain***	4	0.685	0.704	3	0.544	1.73	9.15	1	0.846	0.48	58.33
***Egypt***	6	0.438	0.526	4	0.449	19.50	227.53	1	0.526	15.42	283.17
***Jordan***	1	0.718	0.899	1	0.718	0.03	0.12	0	0.899	0.00	0.89
***Kuwait***	3	0.553	0.565	2	0.580	0.16	3.47	1	0.587	0.09	6.78
***Pakistan***	8	0.620	0.673	1	0.656	212.56	4798.89	1	0.725	168.47	6070.61
***Qatar***	2	0.477	0.554	1	0.520	34.29	281.01	0	0.554	16.49	910.21
***Sri Lanka***	2	0.732	0.762	1	0.770	79.86	1480.44	1	0.770	79.86	1480.44
***United Arab Emirates***	4	0.684	0.801	4	0.684	21.27	104.47	0	0.801	1.64	1166.54
***Bangladesh***	14	0.594	0.652	10	0.601	191.78	1758.09	2	0.653	149.24	3153.59
***Oman***	5	0.773	0.763	4	0.789	0.40	2.50	3	0.791	0.35	5.39
**Average**		**0.627**	**0.690**		**0.631**				**0.715**		
**Total**	**49**			**31**		**561.58**	**8665.67**	**10**		**432.05**	**13135.95**

*Note*: This table provides information on the IBs involved in this study and their efficiency scores for the years 2019 and 2020. The InvDEA approach suggests that banks with drops in any of their outputs need to accordingly revise their inputs used; the InvDEAef approach suggests that only banks with declines in their efficiency scores need to do so. The two inputs are *Operating Expenses* ($x_{1}$) and *Total Deposits* ($x_{2}$), with Δ representing the reduction in the optimal input. Reductions are presented in billion US$ (2010 constant prices).

## 5 Conclusion

This study examined the efficiency and performance of 49 IBs in 10 countries during the COVID‐19 pandemic. Given the adverse impacts of COVID‐19, one would expect to see that some IBs experienced some drops in their operations and performance in 2020, compared with 2019. Therefore, one important question for the managers of IBs is how to maintain the bank's efficiency as good as it was in 2019. Our work proposed a novel InvDEA method based on reduced efficiency (InvDEAef) to answer that question.

Previous studies using InvDEA either used virtual data (e.g., one can virtually merge two individual banks into a new one, with the targeted efficiency being the highest of the two pre‐merged ones) or targeted banks with any adjustments in their inputs or outputs (e.g., if a certain bank changed its outputs, it also needs to adjust the inputs to preserve its efficiency level). Our proposed InvDEAef is an improvement, as we only focus on the banks with reduced efficiency scores. Theoretically, InvDEAef helps narrow down the number of targeted banks, which may be a significant number, considering the size of the banking industry and other sectors, as well as the big data era we live in, which consequently reduces the computational burden of the analysis. More importantly, by focusing on the banks with reduced efficiency, InvDEAef practically helps improve the overall efficiency and performance of the entire sample of banks. It, therefore, sheds new light on the operational management of banks.

The empirical results of our InvDEAef show that, when facing the adverse impacts of COVID‐19, 10 IBs needed to adjust their inputs to maintain their efficiency in 2020 to be as good as it was in 2019. If one uses the traditional InvDEA approach, the figure is 31 IBs. However, even if all 31 IBs adjusted their inputs in 2020, the average efficiency of all 49 IBs in the sample would be even lower than the case of no adjustment (0.631 vs. 0.690). For our proposed InvDEAef approach, the average efficiency of all 49 IBs in 2020 was 0.715, indicating an efficiency improvement, compared with the cases with no adjustment and 31 IBs making adjustments. Consequently, InvDEAef helps the sampled IBs save $432 in operating expenses and $13,136 billion in terms of deposits, which is far better than traditional InvDEA, with $562 billion and $8666 billion in savings for the same inputs, respectively. Given the harsh 2020 and the following years, such cost‐saving advantages have practical importance for bank managers.

Because the InvDEAef is based on DEA and InvDEA, it has the same caveats as those approaches, such as the “curse of dimension,” the sensitivity issue, and the lack of statistical characteristics (Bahrini, 2017; Emrouznejad and Yang, 2018; Ngo and Tsui, 2021). Extensions of InvDEAef, however, would be possible with larger samples and for other industries. These extensions could incorporate more advanced models such as bootstrapping, a common set of weights, weight restrictions, and network DEA (Simar and Wilson, 2007; Tone and Tsutsui, 2009; Hammami et al., 2020; Amin and Ibn Boamah, 2021).

## Footnotes

1 We also notice that for large samples, solving an InvDEAef model could be relatively faster than solving an InvDEA model, as the former should involve fewer banks (or observations) than the latter.
